# Approximating facial expression effects on diagnostic accuracy *via* generative AI in medical genetics

**DOI:** 10.1093/bioinformatics/btae239

**Published:** 2024-06-28

**Authors:** Tanviben Patel, Amna A Othman, Ömer Sümer, Fabio Hellman, Peter Krawitz, Elisabeth André, Molly E Ripper, Chris Fortney, Susan Persky, Ping Hu, Cedrik Tekendo-Ngongang, Suzanna Ledgister Hanchard, Kendall A Flaharty, Rebekah L Waikel, Dat Duong, Benjamin D Solomon

**Affiliations:** Medical Genomics Unit, Medical Genetics Branch, National Human Genome Research Institute, Bethesda, MA 20892, United States; Medical Genomics Unit, Medical Genetics Branch, National Human Genome Research Institute, Bethesda, MA 20892, United States; Institute of Computer Science, Augsburg University, Augsburg, Bavaria 86159, Germany; Institute of Computer Science, Augsburg University, Augsburg, Bavaria 86159, Germany; Institute for Genomic Statistics and Bioinformatics, University of Bonn, Bonn, North Rhine-Westphalia 53113, Germany; Institute of Computer Science, Augsburg University, Augsburg, Bavaria 86159, Germany; Medical Genomics Unit, Medical Genetics Branch, National Human Genome Research Institute, Bethesda, MA 20892, United States; Social and Behavioral Research Branch, National Human Genome Research Institute, Bethesda, MA 20892, United States; Social and Behavioral Research Branch, National Human Genome Research Institute, Bethesda, MA 20892, United States; Medical Genomics Unit, Medical Genetics Branch, National Human Genome Research Institute, Bethesda, MA 20892, United States; Medical Genomics Unit, Medical Genetics Branch, National Human Genome Research Institute, Bethesda, MA 20892, United States; Medical Genomics Unit, Medical Genetics Branch, National Human Genome Research Institute, Bethesda, MA 20892, United States; Medical Genomics Unit, Medical Genetics Branch, National Human Genome Research Institute, Bethesda, MA 20892, United States; Medical Genomics Unit, Medical Genetics Branch, National Human Genome Research Institute, Bethesda, MA 20892, United States; Medical Genomics Unit, Medical Genetics Branch, National Human Genome Research Institute, Bethesda, MA 20892, United States; Medical Genomics Unit, Medical Genetics Branch, National Human Genome Research Institute, Bethesda, MA 20892, United States

## Abstract

Summary

Artificial intelligence (AI) is increasingly used in genomics research and practice, and generative AI has garnered significant recent attention. In clinical applications of generative AI, aspects of the underlying datasets can impact results, and confounders should be studied and mitigated. One example involves the facial expressions of people with genetic conditions. Stereotypically, Williams (WS) and Angelman (AS) syndromes are associated with a “happy” demeanor, including a smiling expression. Clinical geneticists may be more likely to identify these conditions in images of smiling individuals. To study the impact of facial expression, we analyzed publicly available facial images of approximately 3500 individuals with genetic conditions. Using a deep learning (DL) image classifier, we found that WS and AS images with non-smiling expressions had significantly lower prediction probabilities for the correct syndrome labels than those with smiling expressions. This was not seen for 22q11.2 deletion and Noonan syndromes, which are not associated with a smiling expression. To further explore the effect of facial expressions, we computationally altered the facial expressions for these images. We trained HyperStyle, a GAN-inversion technique compatible with StyleGAN2, to determine the vector representations of our images. Then, following the concept of InterfaceGAN, we edited these vectors to recreate the original images in a phenotypically accurate way but with a different facial expression. Through online surveys and an eye-tracking experiment, we examined how altered facial expressions affect the performance of human experts. We overall found that facial expression is associated with diagnostic accuracy variably in different genetic conditions.

## 1 Introduction

Artificial intelligence (AI) applications have made important inroads in many areas of biomedicine, including within the field of medical genetics (Schaefer *et al.*, 2020; Hanchard *et al.*, 2022). One important example in medical genetics involves the use of deep learning (DL) to examine photos of people with suspected genetic conditions to help determine what condition they may have (Hsieh *et al.*, 2022). Despite the individual rarity of most genetic conditions and the fact that datasets are relatively small, leveraging pre-trained models has allowed robust performance (and rapid adoption) of DL-based tools in this context (Duong *et al.*, 2022b). This technological approach may help ameliorate issues like long wait times to see clinical genetics specialists, which arises primarily from a lack of expert clinicians relative to the prevalence of people with genetic conditions (Tekendo-Ngongang *et al.*, 2020; DeGrave *et al.*, 2021; Porras *et al.*, 2021). While these types of DL-based tools have become popular in medical genetics, important questions arise about their performance, especially in the context of rare diseases. As one of many questions, it remains unclear how patient characteristics (e.g. patient age or ancestry, as well as other confounders and potential artifacts) may affect DL model results (Duong *et al.*, 2022a). For example traditional textbooks may depict certain genetic conditions in stereotypical ways (which may not accurately reflect the real-life range of manifestations across affected individuals), and these depictions can bias computational models as well as human clinicians. These types of challenges can lead to delays in the ability to provide precise and accurate genetic diagnoses, which in turn are often necessary for optimal clinical care (Solomon *et al.*, 2013; Incerti *et al.*, 2022).

Human facial expressions represent a complex type of nonverbal communication that is essential for the display of emotions and social cues. Computationally assessing the subtleties of facial expressions has been an area of active research in DL, especially since the introduction of datasets and models such as the Facial Action Coding System (FACS), which has allowed for in-depth and wide-ranging studies (Ekman *et al.*, 1976; Tian *et al.*, 2001; Skiendziel *et al.*, 2019; Rosenberg and Ekman, 2020; Correia-Caeiro *et al.*, 2022). Although many investigations have been performed on facial expressions in general (Li and Deng, 2022; Peña *et al.*, 2021; Zhi *et al.*, 2021; Wehrli *et al.*, 2022), there is still a significant gap in the medical domain, such as how facial expressions may be associated with specific genetic conditions. For example, people with Williams syndrome (WS) and Angelman syndrome (AS) are often portrayed in the medical literature as smiling and/or as having “happy” demeanors (Jones *et al.*, 2021). While these observations may be based on astute clinical observation, this may also mean that diagnoses may be missed when a patient with one of these conditions is not smiling when assessed, or otherwise exhibits atypical characteristics (Tanzi and Bertram, 2001; Richmond *et al.*, 2018). Our previous work (Duong *et al.*, 2022a) suggested that clinician performance changes significantly with respect to the facial expressions of images of people with WS and 22q11.2 deletion syndrome (22q). For example, with WS, we found that clinicians were more likely to correctly suspect this condition for those patients with a smiling expression. We did not observe a similar phenomenon with 22q (see details in Supplementary Table 1). This is expected based on textbook explanations of these conditions, but as described above, can be a barrier to efficient diagnosis and appropriate medical care. As individuals are often diagnosed in medical situations, such as through a visit to a clinician or an inpatient admission, clinicians in our group (and those with whom we have discussed this question) have anecdotally reported that patients may be less prone to smiling in such scenarios, which may affect clinicians’ diagnostic assessments in light of what is expected based on textbook descriptions.

Following our preliminary observations, we wanted to expand this investigation by including other conditions besides WS and 22q. Moreover, since the above-mentioned cohorts, which were divided based on “smile versus no smile” expressions, were made up of different individuals, it is possible that one group of individuals was simply easier to identify *via* other syndromic factors besides the face expressions. Hence, in this article, we further conducted the experiments in a manner that allows us to better control for these potential confounders.

Specifically, we used Generative Adversarial Network inversion (GAN-inversion) technique to systematically modify the facial expressions of real individuals. Next, we assessed how the altered facial expressions affect the performance of a DL classifier and human clinicians. For human clinician assessment, we employed web-based surveys to evaluate how well clinicians identify individuals with genetic conditions, and eye-tracking experiments, which allow us to observe human visual attention with respect to specific facial regions in images of people with genetic conditions. We anticipate that these results can demonstrate how a specific confounder can influence a DL classifier and human clinicians in the assessment medical conditions. We hope this work can contribute to improved diagnosis and management of people and families affected by these disorders.

## 2 Methods

### 2.1 Data collection

Following our previous work (Duong *et al.*, 2022a, 2022b) we identified and used publicly available images depicting individuals with four conditions: 22q (OMIM #611867), AS (OMIM #105830), Noonan syndrome (NS) (OMIM #163950, 605275, 609942, 610733, 611553, 613706, 615355, 616559, 616564, 619087), and WS (OMIM #194050). We selected these genetic conditions because they have recognizable facial features and are rare but still occur relatively frequently compared to many other genetic conditions. Additionally, we chose AS and WS because of the “textbook definitions” of the facial expressions of affected individuals. For example, individuals with AS or WS are often described as having a happy demeanor or a smiling expression, whereas individuals with 22q and NS are not typically depicted this way (Jones *et al.*, 2021).

For each image, we documented reported age and gender when this information was provided by the source. We sought images of individuals with diverse ancestry, intentionally aiming for diverse representation. However, as the genomic ancestry details were not always available for all individuals, we did not want to assume a person’s specific genomic ancestry if not clearly provided.

We also used images of individuals with other genetic conditions to train the ResNet-50 image classifier and GAN image generator (see condition names in Table 1). These other conditions may exhibit similar syndromic features to our diseases of interest. Hence, having images of these other conditions may help us better classify and generate images of 22q, AS, NS, and WS.

**Table 1.** Fivefold cross-validation performance of ResNet-50 based on the 3484 training images (excluding the 32 test images in the clinician survey and eye-tracking experiment).

	Precision	Recall	F1-score	Sample size
22q	0.7198	0.8256	0.7691	585
AS	0.7847	0.7969	0.7908	453
BWS	0.6974	0.6156	0.6539	307
CdLS	0.7500	0.7804	0.7649	123
Down	0.8591	0.8615	0.8603	354
KS	0.8302	0.7364	0.7804	239
NS	0.7454	0.7620	0.7536	269
PWS	0.6039	0.5700	0.5865	107
RSTS1	0.8152	0.6944	0.7500	108
Unaffected	0.6619	0.5840	0.6205	238
WHS	0.7446	0.7865	0.7650	178
WS	0.8790	0.8757	0.8773	523
Average	0.7576	0.7408	0.7477	3484

Accuracy metrics are averaged across the five folds. The average top-1 accuracy over all the diseases is 0.7715 (not shown table).

Abbreviations: 22q, 22q11.2 deletion syndrome; AS, Angelman syndrome; BWS, Beckwith–Wiedemann syndrome; CdLS, Cornelia de Lange syndrome; Down, Down syndrome; KS, Kabuki syndrome; NS, Noonan syndrome; PWS, Prader–Willi syndrome; RSTS1, Rubinstein–Taybi syndrome type 1; WHS, Wolf–Hirschhorn syndrome; WS, Williams syndrome.

In total, we collected 3528 facial images of individuals affected with 11 genetic conditions, as well as images of unaffected individuals, including 590 22q images, 456 AS images, 327 NS images, and 529 WS images. We set aside 32 images (eight for each of these four genetic conditions) so that we could evaluate these images in the human assessment experiments. These 32 test images were chosen showing individuals from 2 to 20 years of age since optimal medical management depends on early clinical diagnosis, and as these conditions are all recognizable in childhood.

Twelve images were excluded due to lack of recognizable facial landmarks. This results in a dataset of 3484 (3528 minus 32 human test images and 12 images with unrecognizable landmarks) for training ResNet-50.

Our GAN image generator was trained with 3516 images (3528 minus 12 images with unrecognizable landmarks). We note that the 3516 includes the 32 test images. Our key objective was to train the image generator on these test images. This allows us to generate high-quality (i.e. realistic and clinically accurate) versions of these test images with different facial expressions.

To summarize, images in this study were either (1) the real images, (2) the reconstructed versions of these real images done *via* GAN-inversion, which we will refer to as “reconstructed images” (see Section GAN-inversion), and (3) the reconstructed images with modified facial expressions which we will refer to as “expression-manipulated images”. Reconstructed images primarily depicted individuals with 22q, AS, NS, or WS having the typical facial expressions of these syndromes (i.e. AS and WS with a smiling expression and 22q and NS with a non-smiling expression). Expression-manipulated images were computationally altered to have the opposite facial expressions (i.e. AS and WS with a non-smiling expression and 22q and NS with a smiling expression).

### 2.2 Syndromic image classifier

To classify the images with respect to their genetic conditions, we based our ResNet-50 classifier on the approach of (Sümer *et al.*, 2023). This ResNet-50 classifier was pre-trained on the VGG Face-2 dataset (Cao *et al.*, 2018b; Krawitz *et al.*, 2021; Sümer *et al.*, 2023). Our training dataset size for the fivefold cross-validation is 3484 images (as explained in the Data Collection section of the Methods).

For data preprocessing, all images were rescaled to 224 × 224 pixel resolution. Next, we followed the StyleGAN2 approach to standardize image sizes and facial landmark alignment (Abdal *et al.*, 2019). We selected StyleGAN2 data preprocessing because it was also used by the GAN-inversion approach HyperStyle (see next section).

For the fivefold cross-validation, we trained and evaluated ResNet-50 five times, each time using a different fold as the validation set and the remaining four folds as the training set. We followed the hyperparameter setting in Sümer *et al.* (2023), and used 32 batch size and SGD optimizer with learning rate 0.001, momentum 0.9, and Cosine annealing warm start. We trained for 35 epochs (about 5 h on one p100 GPU) and selected the saved model with the best validation loss out of these epochs.

For a test image, the predicted probability is computed by averaging the outcome across all folds. The training and inference script for this ResNet-50 is available at our GitHub (https://github.com/pateltanvi2992/genetic-conditions-image-classifier.).

### 2.3 GAN-inversion and facial expression manipulation

A typical GAN image generator maps an input vector (usually randomly drawn from a normal distribution) into a new output image that looks realistic with respect to the training dataset. To reverse the process of the image generator, given an image of interest, one would need to solve for the vector that can recreate an output appearing very similar to this image of interest. We note that this image of interest can be either a fake or real image. This reverse process is known as GAN-inversion and its output is called the “reconstructed image” of the original image of interest (Alaluf *et al.*, 2022). By solving for the vector that can be used to reconstruct the input image, GAN-inversion allows us to edit this image by manipulating its corresponding vector representation.

StyleGAN is one of several GAN architectures for creating high-quality synthetic images, including the ability to generate realistic and diverse human faces. Although StyleGAN can generate fake images, it cannot reliably change the face expressions of real images. We opted for the GAN-inversion approach HyperStyle, which has two main advantages over StyleGAN: (1) HyperStyle uses a hypernetwork to efficiently update StyleGAN weights according to our unique datasets, and (2) HyperStyle can edit the facial expressions of real images (Abdal *et al.*, 2019; Von Oswald *et al.*, 2019; Alaluf *et al.*, 2022).

For human faces, almost all GAN-like methods are trained with datasets like Flickr-Faces-HQ (FFHQ) or CelebFaces Attribute (CelebA) (Guo *et al.*, 2016; Cao *et al.*, 2018a; Karras *et al.*, 2019; Zhao *et al.*, 2020). The original authors of HyperStyle trained their model on FFHQ, which does not contain faces of individuals affected with the genetic conditions as in our analysis. Hence, we could not reliably apply their pre-trained HyperStyle to reconstruct the images in our dataset. Initially, we finetuned HyperStyle on just our small dataset of 3516 images (see Data Collection section), but as this sample size is too small, finetuning with various hyperparameter options did not provide high-quality outputs.

For this reason, we combined our dataset with 70 000 FFHQ images. Specifically, we first randomly partitioned the 70 000 FFHQ images into 59 600 and 10 400 for training and validation, respectively. Next, we added our 3516 syndromic images into the training set. This yields a final training dataset of size 63 116 (from 59 600 and 3516) which enabled us to reliably train HyperStyle. Next, we followed the exact hyperparameter settings as described by the original authors of HyperStyle and trained their model from-scratch on the combined dataset. Our GitHub (https://github.com/pateltanvi2992/Analyzing-Facial-Expressions-with-Generative-AI-in-Clinical-Genetics/.). provides the training and inference script to reproduce the results in this article. Here, we briefly describe a few key hyperparameters. Following the setting in Alaluf *et al.* (2022) we used batch size 8 and the ranger optimizer (Wright, 2019) with learning rate 0.0001. We trained until the validation loss converged, which took 109 000 epochs and 10 days on one v100x GPU.

**Figure 1.**
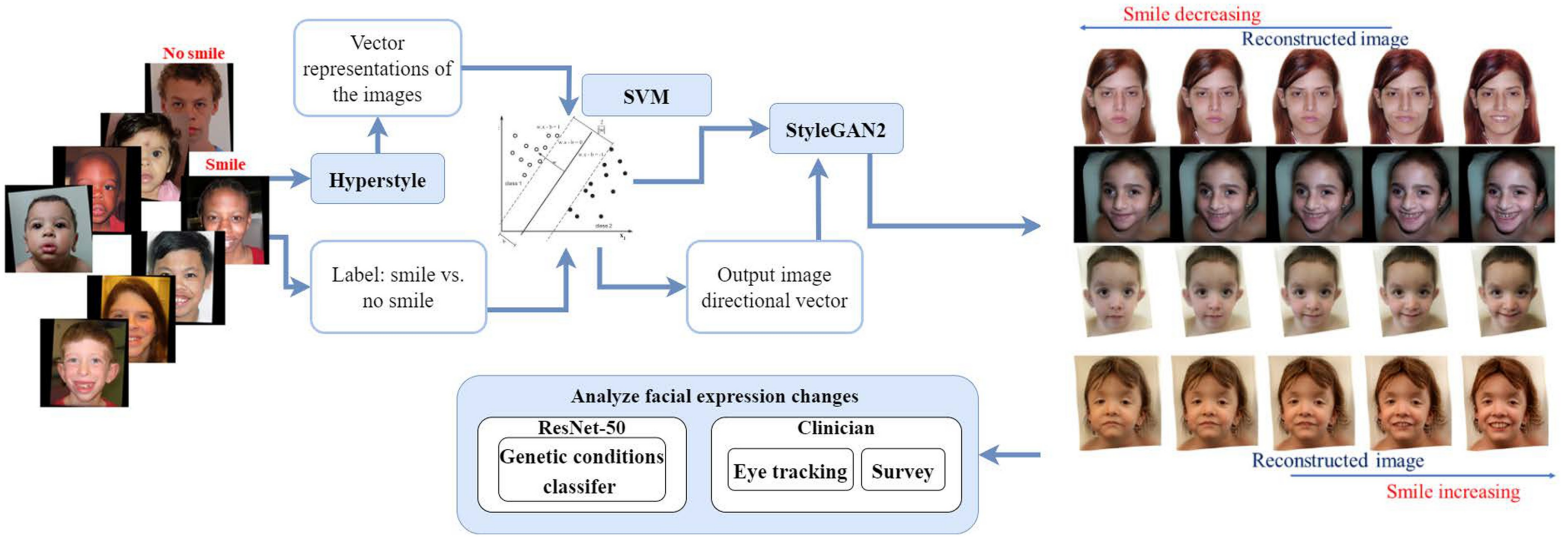
Manipulating facial expressions in images of people with genetic conditions *via* HyperStyle and StyleGAN2. We started with our real images, and manually classified these facial expressions into smile versus no smile. We finetuned HyperStyle, where the goal is to find an image embedding that can reconstruct the original input image (*via* StyleGAN2). Next, we manipulated the embedding of the reconstructed image by using SVM as shown in InterfaceGAN (Pisner and Schnyer, 2020). Finally, we passed the newly edited embedding into StyleGAN2 to create a new image with a new facial expression that still closely resembles the original input. On the far right, the examples illustrate the conditions we analyzed. All appropriate permissions have been obtained to reuse images and the citations for each are provided in Supplementary Table 5.

During the inference phase, we applied HyperStyle to obtain the vector representations of the original images, and then manipulated these vectors to create new face expressions for the original images (see Figure 1 and Supplementary Figure 1). That is, in the first step, we solve for the vector representations that can generate outputs (i.e. the reconstructed images) that look like the original images. In the second step, we manipulated the facial expressions of the reconstructed images to produce the expression-manipulated outputs.

To alter the face expressions, we followed the concept of InterfaceGAN (Shen *et al.*, 2020). After obtaining the vector representations of the reconstructed images, we applied Support Vector Machine (SVM) to approximate the “direction vector” that separates the “boundaries” between images with and without smiling expressions. This direction vector allowed us to change the facial expressions by modifying the vector representations of the reconstructed images; this step produces the expression-manipulated images. For each of the four genetic conditions, we found their own unique direction vector *via* their own unique SVM (Figure 1).

Subjectively, our own clinicians deemed the reconstructed images and their corresponding expression-manipulated outputs to be realistic representations of the given conditions. We further verified these reconstructed images and their expression-manipulated versions *via* an independent classifier built to analyze genetic conditions (Hsieh *et al.*, 2022). This is an important validation as the presence of a genetic condition can influence the appearance of facial expressions beyond whether a person is smiling or not. However, reconstruction may still introduce undetected changes that may affect the ability of clinicians to recognize the conditions.

### 2.4 Clinician surveys and eye-tracking experiments

After generating expression-manipulated syndromic images, we compared the accuracy of human experts (clinical geneticists) on the reconstructions of the original images versus the expression-manipulated images *via* electronic surveys sent using Qualtrics (Provo, Utah, United States). Using the reconstructions of the original images would include GAN artifacts, which would also be found in the expression-manipulated images. Hence, this would allow for a fair comparison between smile and no smile images. We used two versions of the survey, allowing for a reconstructed image to be shown to one group and the corresponding expression-manipulated image to be shown to the other group. Each survey included 32 total images, with an equal number of reconstructed and expression-manipulated images. Participants were asked to classify each image as showing a person with either 22q, AS, NS, WS, or who was unaffected by a genetic condition. While there were no unaffected images included in the test set, we included this option in our surveys to investigate whether expression affected the participants’ perceptions of whether the image showed a person affected by a genetic condition or not. Example surveys are available at our GitHub (https://github.com/pateltanvi2992/Analyzing-Facial-Expressions-with-Generative-AI-in-Clinical-Genetics/tree/main/survey/.).

Participants were recruited *via* email. To identify survey respondents, we obtained email addresses through professional networks, departmental websites, journal publications, and other publicly available lists.

We also conducted a visual attention experiment involving images of individuals whose facial images were first reconstructed and then expression-manipulated to study how clinicians observe facial expressions. Our objective was to investigate whether clinicians’ visual attention was affected by changes in facial expression for individuals with genetic conditions. This experiment also extends prior research, highlighting distinctions in the assessment of genetic conditions between human observers and DL models (Bours *et al.*, 2018).

For this part of our study, the formatted images were embedded in a screen-based eye-tracking system (Tobii Pro X3-120, Stockholm, Sweden). To examine the effects of expression changes on general clinicians, we recruited 10 clinicians at the National Institutes of Health (NIH) in Bethesda, Maryland, United States to take part in the eye-tracking experiments. Participants were instructed to view the images as if assessing patients in a clinic.

After calibration, each participant viewed the 32 images for 7 s per image. We chose 7 s for the viewing time after extensive initial testing and per our previous work using eye-tracking in a related way, as subjective feedback and preliminary assessments showed that this amount of time was sufficient to assess an image but minimized participants visually revisiting areas of the image in a way that might not inform the assessment (Duong *et al.*, 2023).

**Figure 2.**
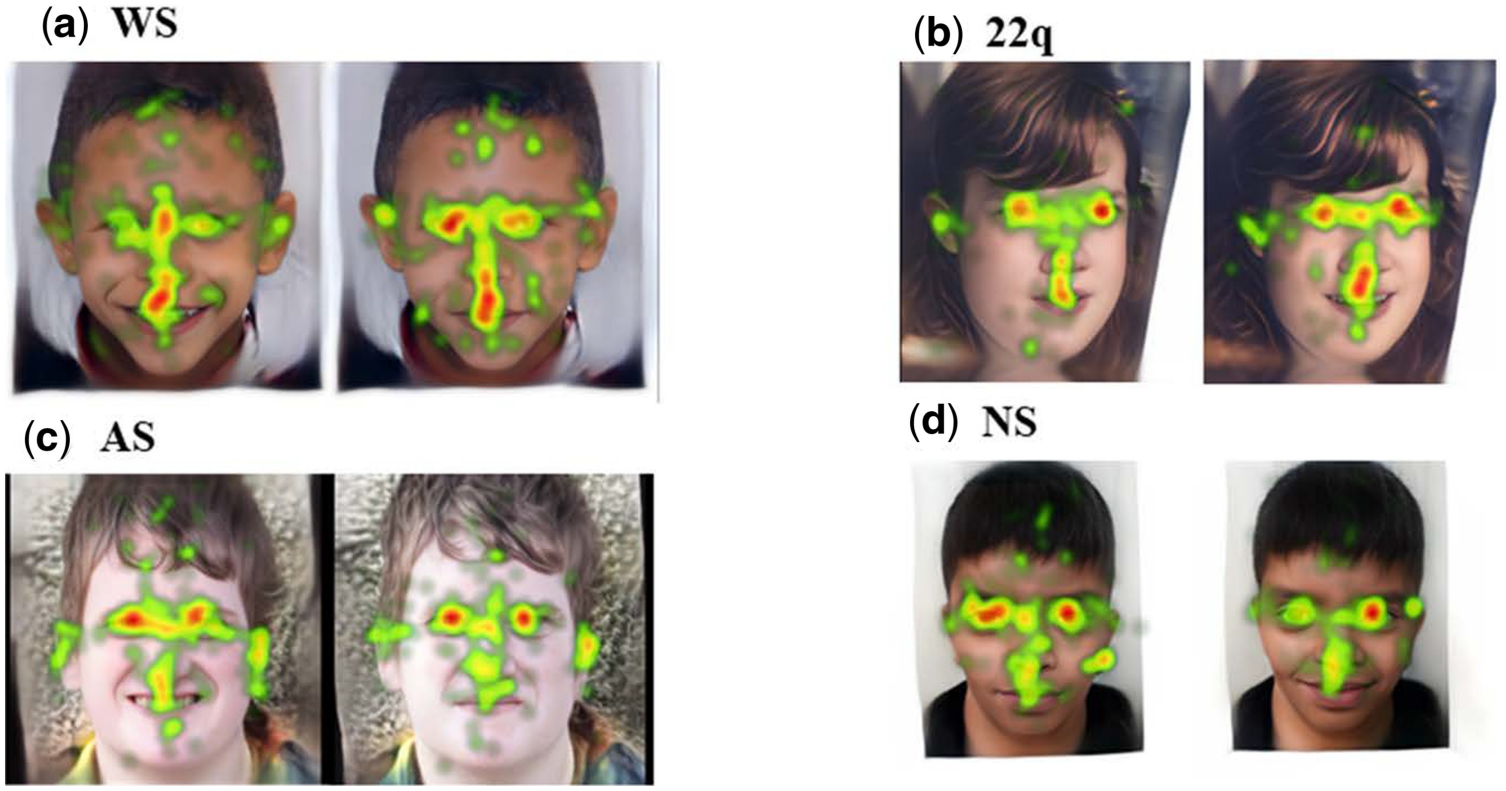
Example outputs of the eye-tracking experiment. Each image illustrates the visual heatmap averaged over 10 clinicians. Each set of images includes the reconstructed image (left) and its corresponding expression-manipulated version (right). All appropriate permissions have been obtained to reuse images and the citations for each are provided in Supplementary Table 5.

At the end of the experiment, we analyzed the amount of time the participants looked at the custom area-of-interest (AOI) drawn around the mouth. This would approximate the effect of the facial expressions on human visual attention. To minimize bias, we used the same AOI for each image (see examples in Figure 2).

The study was formally approved as IRB exempt by the NIH IRB [IRB# 001686 (clinical geneticist survey), 001684 (clinician eye-tracking)].

## 3 Results

### 3.1 Model performance on validation set

Table 1 shows the overall performance of ResNet-50 at identifying each of the genetic conditions on the validation set; the metrics are averaged over five folds. This result indicates that our trained ResNet-50 can adequately recognize our disease of interests: 22q, AS, NS, and WS.

Next, we assessed ResNet-50 performance with respect to the facial expressions that were manually annotated by our clinicians (Table 2).

**Table 2.** Measuring the effects of facial expression on classifier accuracy.

	Expression	No. of images	Prediction probability	*P*-value	Top-1 accuracy	*P*-value
22q (not associated with smile)	No smile	425	0.8247	0.1457	0.8400	0.1362
	Smile	160	0.7778		0.7875	
AS (associated with smile)	No smile	111	0.6481	$3.19\times 10^{-5}$	0.6937	$1.47\times 10^{-3}$
	Smile	346	0.8128		0.8324	
NS (not associated with smile)	No smile	226	0.7847	0.0709	0.7965	0.0322
	Smile	39	0.6670		0.6410	
WS (associated with smile)	No smile	276	0.8227	$6.94\times 10^{-4}$	0.8333	$1.85\times 10^{-3}$
	Smile	247	0.9156		0.9231	

Our facial image dataset was manually labeled as “smile” or “no smile”. We assessed the classifier performance when assessing expression-labeled images, which we refer to as the “average prediction probability”. Additionally, we assess the overall performance of the model in accurately classifying the primary category, which we refer to as “average top-1 accuracy”. WS and AS are classically associated with smile, while 22q and NS are not.

Besides using the manually labels for facial expressions, we also automatically measured the smile intensity using the FACS (Rosenberg and Ekman, 2020). Since FACS is a systematic approach, it may capture more subtle changes (e.g. continuous or incremental changes) in facial expression than a human labeler may report (e.g. binary label of smile versus not smiling). Regardless of using human labeling or FACS, we still expect that there should be a correlation between diagnostic accuracy and facial expressions for most of the four chosen conditions.

**Figure 3.**
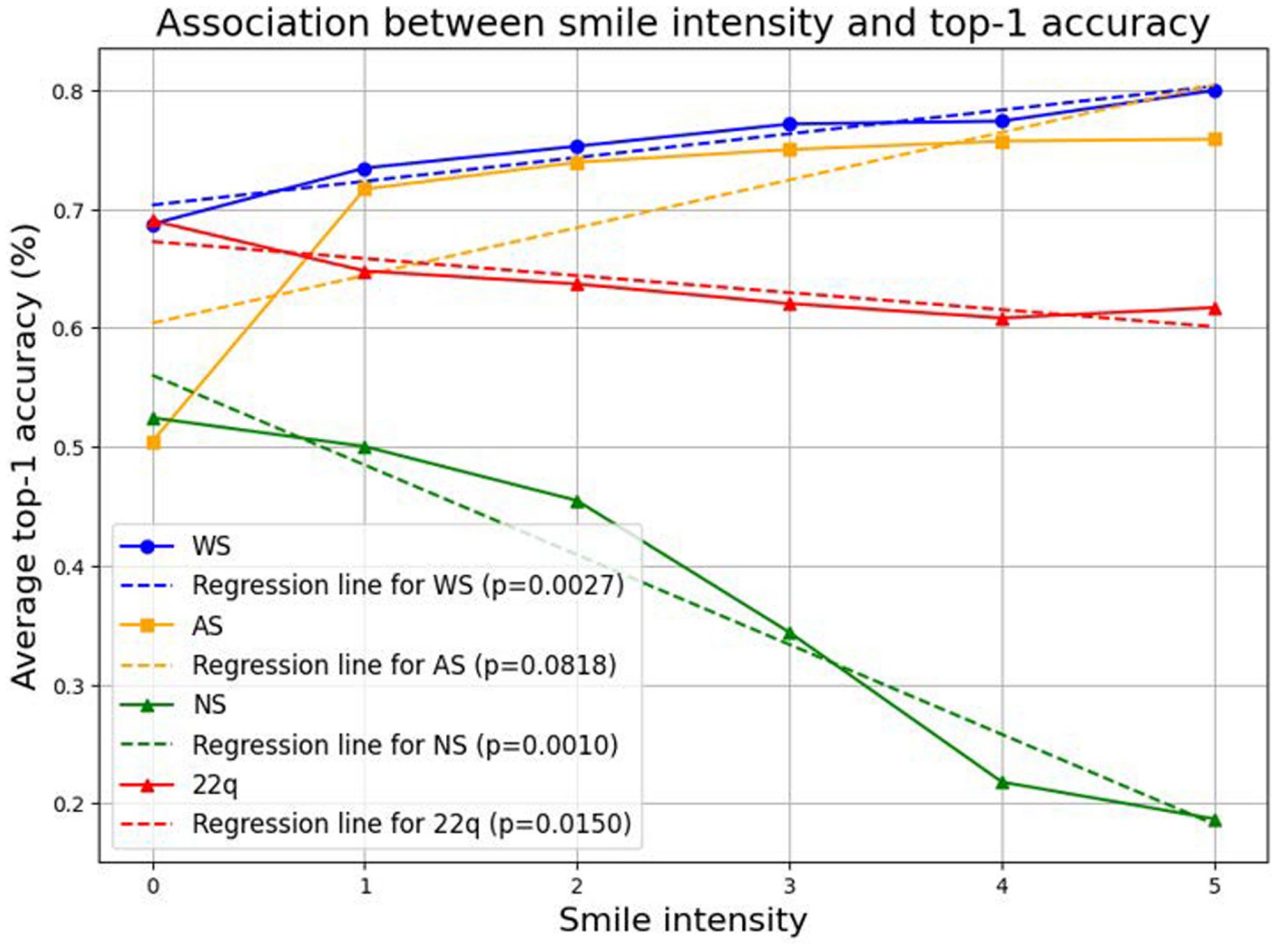
Top-1 accuracy with respect to smile intensity as determined by Facial Action Coding System (FACS). Smile intensity was calculated for each facial image by adding facial action units 6 (cheek raiser) to 12 (lip corner puller). Top-1 accuracy refers to the ability of the classifier to predict the correct genetic condition (highest prediction probability). With higher smile intensity, there is a considerable decrease in correct prediction of top-1 accuracy in NS and a more modest decrease in 22q. Conversely, there is an increase in top-1 accuracy in AS and WS with greater smile intensity.

We first selected images that our ResNet-50 classifier correctly identified *via* top-1 accuracy. Then, we measured smile intensities *via* FACS, focusing on AU6 (Action Unit: Cheek Raiser) and AU12 (Action Unit: Lip Corner Puller), which together indicate a genuine and spontaneous smile (Tian *et al.*, 2001). Overall, in the context of FACS measurement, we observed significant changes in disease classification accuracy with respect to the facial expression in WS, NS, and 22q (Figure 3).

Figure 3 shows significant changes in top-1 accuracy for WS (P=0.0027) and NS (P=0.0010) with respect to smile intensity. This result agrees with Table 2, which shows significant changes for WS and NS ($P=1.85\times 10^{-3}$) and (P=0.0322), respectively. Interestingly, for AS, there is a significant difference observed in Table 2 ($P=1.47\times 10^{-3}$) but not in Figure 3, as the *P*-value for the association between accuracy and smile intensity is 0.0818. However, in AS the accuracy increases slightly after the first smile intensity level, with the most significant improvement happening during the transition between smile intensity 0 and 1. For 22q, Table 2 does not show a significant difference (P=0.1362) while Figure 3 does (P=0.0150). Overall, there are no major contradictory outcomes between FACS and our human labeling, except for slight differences in AS and 22q.

### 3.2 Model performance on reconstructed and expression-manipulated test images

Our test dataset consisted of 32 images, with eight images from each of the following categories: WS, NS, AS, and 22q. These images were also used to evaluate the performance of human clinicians (see below). We evaluated the classifier performance on the reconstructed images and expression-manipulated images of these 32 real images.

All reconstructed images of WS, 22q, and NS were correctly identified by the classifier. In the case of AS, two incorrect predictions were made, with prediction probabilities of 33.59% and 41.34% for the correct disease label, respectively. Table 3 illustrates the comparison between the average accuracies of reconstructed and expression-manipulated images. We observed significant changes in accuracies when altering facial expression for different genetic conditions. For 22q and NS, classifier accuracy dropped when changing the expression to a smile. Conversely, accuracy for AS decreased when transitioning from smile to no smile. However, the accuracy in WS remained consistently high, changing only slightly with expression-manipulated (P=0.3442).

**Table 3.** Model performance on reconstructed and expression-manipulated test images.

	Expression	No. of images	Average ground truth prediction probability	*P*-value
22q (not associated with smile)	Reconstructed	8	0.9420	0.0098
	Smile manipulated	8	0.8452	
AS (associated with smile)	Reconstructed	8	0.7254	0.0236
	No smile manipulated	8	0.5969	
NS (not associated with smile)	Reconstructed	8	0.8788	0.0353
	Smile manipulated	8		
WS (associated with smile)	Reconstructed	8	0.9836	0.3442
	No smile manipulated	8	0.9878	

A total of 32 images were included in the experiments conducted with human clinicians, with eight images from each of the 22q, AS, NS, and WS categories. The first column indicates the classically expected expression (or lack thereof) for each condition. The second column indicates that the expression was manipulated to the opposite expression. For example, AS is associated with smile, so the expression manipulated images were changed to no smile. The expression-manipulated images showed a decrease in accuracy for the 22q, AS, and NS image groups.

### 3.3 Clinician performance on reconstructed and expression-manipulated test images

A total of 314 clinical geneticists were contacted and asked to complete the surveys. In response, 56 surveys were completed (17.8% total response rate), with 23 and 33 surveys completed for the two survey versions, respectively. A survey was considered complete if all survey questions were answered and the survey was submitted. See Supplemental Table 2 for a description of survey respondents.

**Figure 4.**
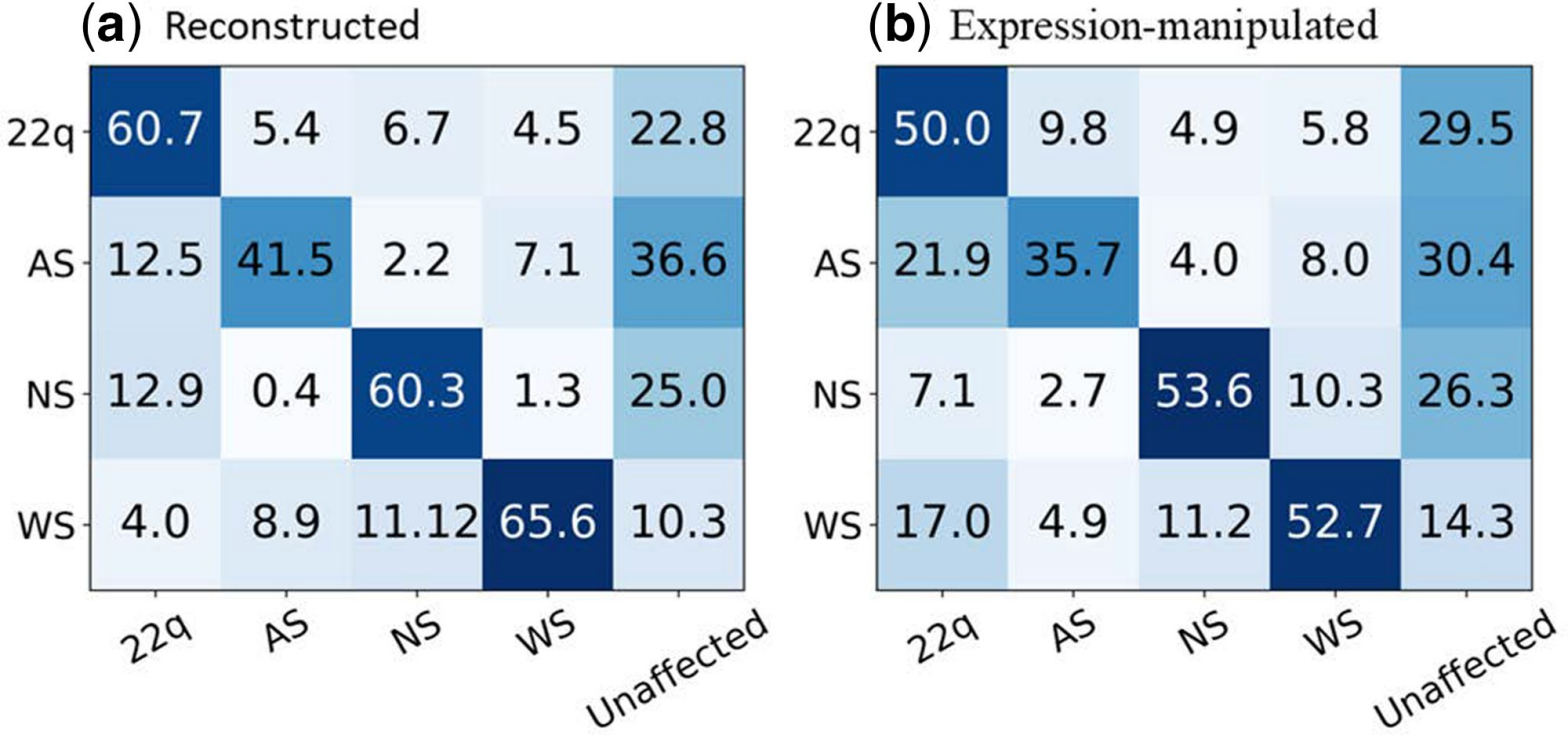
Confusion matrices for the reconstructed (a) and expression-manipulated images (b) based on the performances of the clinical geneticists. In total, 56 clinical geneticists classified 32 images with one of five labels (classification was done *via* a Qualtrics survey).

The average accuracies for the two surveys were 53.3% and 52.0%, which were not statistically different (*P* = 0.575) (see Supplementary Table 3). Both surveys were combined to determine the diagnostic accuracy for each of the four conditions (i.e. the accuracies for the reconstructed images and their expression-manipulated versions). For all conditions, clinician performance decreased for the images manipulated to have the opposite facial expression with respect to their classically expected representation (Table 4). The largest differences were observed in 22q and WS. When non-smiling 22q images were manipulated to show a smiling facial expression, accuracy decreased from 60.7% to 50% (P=0.022). When smiling WS images were manipulated to a more neutral facial expression, accuracy decreased from 65.6% to 52.7% (P=0.005) (Table 4). Unlike the model, the average clinician performance did not statistically decrease in AS and NS. Figure 4 shows the confusion matrices averaged over all the participants who saw the reconstructed images and their expression-manipulated versions, respectively. See Supplemental Table 4 for the correlations between the performance of the classifier and clinical geneticists.

**Table 4.** Clinical geneticist performance at classifying reconstructed and expression-manipulated images.

	Overall accuracy (%)	Average accuracy for reconstructed images (%)	Average accuracy for expression-manipulated images (%)	*P*-value
22q	55.40	60.70	50	0.0225
AS	38.50	41.30	35.70	0.2295
NS	56.90	60.30	53.60	0.1531
WS	59.15	65.60	52.70	0.005

A total of 56 clinical geneticists participated in the survey. See also Supplemental Table 2.

For the eye-tracking experiment, over all the images, there was not a significant difference for the visual attention to the AOI between smile and no smile groups (P=0.4445). There was also not a significant difference for each individual condition, though we observed a small, almost significant trend for WS (P=0.0550), which aligns with the largest diagnostic accuracy drop in Table 4 (65.60% versus 52.70%, P=0.005, between reconstructed versus expression-manipulated images). For each condition, results of smile versus no smile were as follows: 22q: P=0.3629; AS: P=0.4605; NS: P=0.6231; WS: P=0.0550.

## 4 Discussion

Our analyses approximate how changing specific variables like facial expression affects the identification of individuals with 22q, AS, NS, and WS (Oliver *et al.*, 2007). For the ResNet-50 classifier, we observed a significantly higher prediction probability for the ground-truth labels in AS and WS with smiling versus non-smiling expression, but did not observe a significant difference for NS or 22q.

Since the groups of smiling and non-smiling images are not of the same individuals, there can be bias if syndromic features of one group can be more easily recognized. Thus, we next employed HyperStyle, which enabled us to alter facial expressions of the real images while maintaining high fidelity with respect to the genetic conditions (i.e. ensuring the edited image still retained features specific to a particular genetic condition). Although there are other recent tools to manipulate images (Ramesh *et al.*, 2021; Rombach *et al.*, 2022), which can be amusing and impressive for creative undertakings, they do not reliably produce medically accurate images.

For the expression-manipulated images, ResNet-50 accuracy drops for AS when the expression changed from smile to no smile. Similarly, the accuracy dropped when NS and 22q image expressions were changed from no smile to smile. In addition to the limitations of our study (see below), there may be intrinsic differences in the ability to identify WS. That is, people with WS may have additional features that make this condition easier to identify regardless of facial expression.

Besides evaluating the DL image classifier, we also investigated how facial expression affects the performance of human clinicians. Unlike the classifier, we found that expression changes had a significant effect on the clinical geneticists when assessing WS but not AS. We did not observe any major differences in clinician visual attention to the facial regions encompassing the smile (i.e. the areas around the mouth). Overall, participants spend approximately the same amount of time viewing the mouth area with or without a smiling expression. However, the change in accuracy that we observed implies that other features besides the mouth contribute to diagnostic accuracy. These results suggest that there may be key syndromic features associated with smiling rather than simply the mouth expression alone. For example smiling may affect the appearance of the eyes or other parts of the face, and this may occur in a different way for different genetic conditions. This conclusion stems from the fact that we used the same set of patient images with and without the smiling expression (i.e. the same patient with an edited face expression). This inference would not be possible if the two sets of tested images were of different patients. We note that this is an approximation, since the survey-based participants and the eye-tracking participants are not the same individuals and were intentionally chosen to represent different types of clinicians with differing familiarity with genetic conditions (clinical geneticists for the surveys and general clinicians for the eye-tracking experiments).

Due to our small sample size, we could not further assess differences besides the mouth expression in the eye-tracking experiments, but future work could be done to further determine which other regions of the face contribute to classifier and human accuracy with different facial expressions. For example, future experiments to further test the effects of expression changes *via* eye-tracking could involve repeating the eye-tracking experiments with the area of the mouth masked.

Related to these points, our study has multiple limitations. Due to the small number of images in this current study (in both the training and testing set) our future work would focus on collecting more images of different racial, ethnic, age, and gender groups, and of many other genetic conditions (Kruszka *et al.*, 2017; Tekendo-Ngongang *et al.*, 2020; Duong *et al.*, 2022b; Kruszka and Tekendo-Ngongang, 2023; Solomon *et al.*, 2023). Our current dataset was also not perfectly balanced with respect to different types of facial expressions. For example, there was a noticeable disparity in expression in the NS images, with fewer smiling versus non-smiling faces. Thus, while we find our results intriguing, they require additional inquiry using larger datasets. A larger and more diverse dataset could also ensure that generative AI methods work equitably across different populations.

Additionally, our choice of HyperStyle may fail for images that were not seen during training. This was our rationale for including the images we used for human testing into the training set for HyperStyle. Future studies will focus on how to efficiently build generative models to edit new, unseen images. We note that there are other image editing approaches (e.g. diffusion-based methods), and planned work would include evaluating these other techniques (Rombach *et al.*, 2022).

Another limitation was that we could only recruit a limited number of clinicians. In future studies, it may be interesting to further assess how different facial expressions affect non-expert geneticist clinicians, who may be less familiar with the overall range of presentation of genetic conditions, but who may more frequently encounter these patients due to the dearth of clinical genetics experts.

Despite these limitations, one overall interesting conclusion, which echoes some of our previous work, is that a DL model and humans simply perform differently under different circumstances (Duong *et al.*, 2023). As AI tools are adopted in clinical scenarios, better understanding of these differences will be important to ensure that they are useful in helping to diagnose and care for people affected by genetic conditions.

We predict that generative AI tools will become increasingly used in medical settings (Waikel *et al.*, 2023). We hope that the use of these tools can be helpful to clinicians and patients in many situations. In genetics, these approaches may help diagnose patients more quickly and cost-effectively, perhaps especially in underserved communities and parts of the world where there is less access to subspecialists who are very familiar with rare genetic conditions (Solomon *et al.*, 2023). However, we emphasize that it is critical to carefully study DL tools to better understand their strengths and limitations. Even before embarking on clinical implementation studies, it will be important to understand ways to maximize benefits and minimize risks.

## Supplementary Material

btae239_Supplementary_Data

## Data Availability

The data and code underlying this article are available in the article and supplementary materials, with links to code and additional data sources provided in online supplementary material.
