# Correction: Approximating facial expression effects on diagnostic accuracy *via* generative AI in medical genetics

**DOI:** 10.1093/bioinformatics/btag651

**Published:** 2026-09-02

This is a correction to: Tanviben Patel, Amna A Othman, Ömer Sümer, Fabio Hellman, Peter Krawitz, Elisabeth André, Molly E Ripper, Chris Fortney, Susan Persky, Ping Hu, Cedrik Tekendo-Ngongang, Suzanna Ledgister Hanchard, Kendall A Flaharty, Rebekah L Waikel, Dat Duong, Benjamin D Solomon, Approximating facial expression effects on diagnostic accuracy *via* generative AI in medical genetics, *Bioinformatics*, Volume 40, Issue Supplement_1, July 2024, Pages i110–i118, https://doi.org/10.1093/bioinformatics/btae239

The last name of the fourth author was errored in the original publication and the name should read: “Fabio Hellmann”.

The error is outlined only in this correction notice to preserve the version of record.

