# Supplementary material for: Approximating facial expression effects on diagnostic accuracy via generative AI in medical genetics
Source: Bioinformatics. 2024 Jun 28;40(Suppl 1):i110–8. doi: 10.1093/bioinformatics/btae239 (PMC11211818; doi:10.1093/bioinformatics/btae239)
Supplement: btae239_Supplementary_Data [file btae239_supplementary_data.pdf]

Supplementary Figures and Tables

(a)

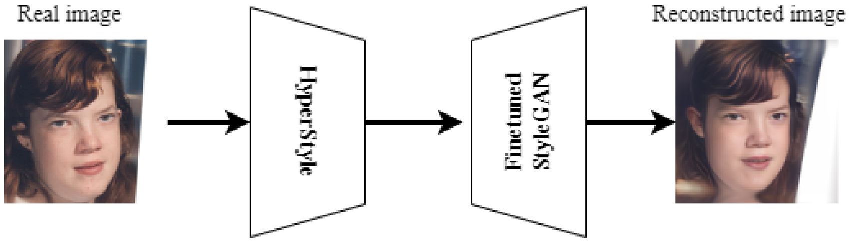

(b)

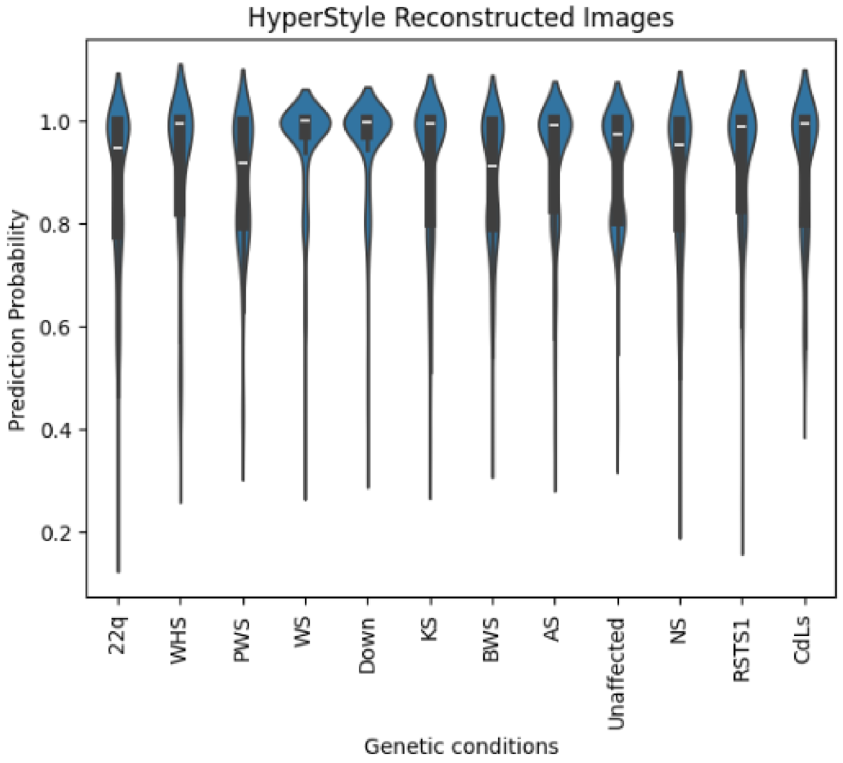

Supplementary Figure 1 : Classifier results on reconstructed images using HyperStyle. Reconstructed images were generated after training HyperStyle on our dataset combined with FFHQ dataset. (a) HyperStyle was applied to solve for the vector that can be used to reconstruct the original image. This vector was then passed through a finetuned StyleGAN (which is also part of the HyperStyle pipeline) to finally produce a reconstructed image. As shown in (b), the classifier assessed the reconstructed images. Most reconstructed images were accurately classified with respect to their labels.

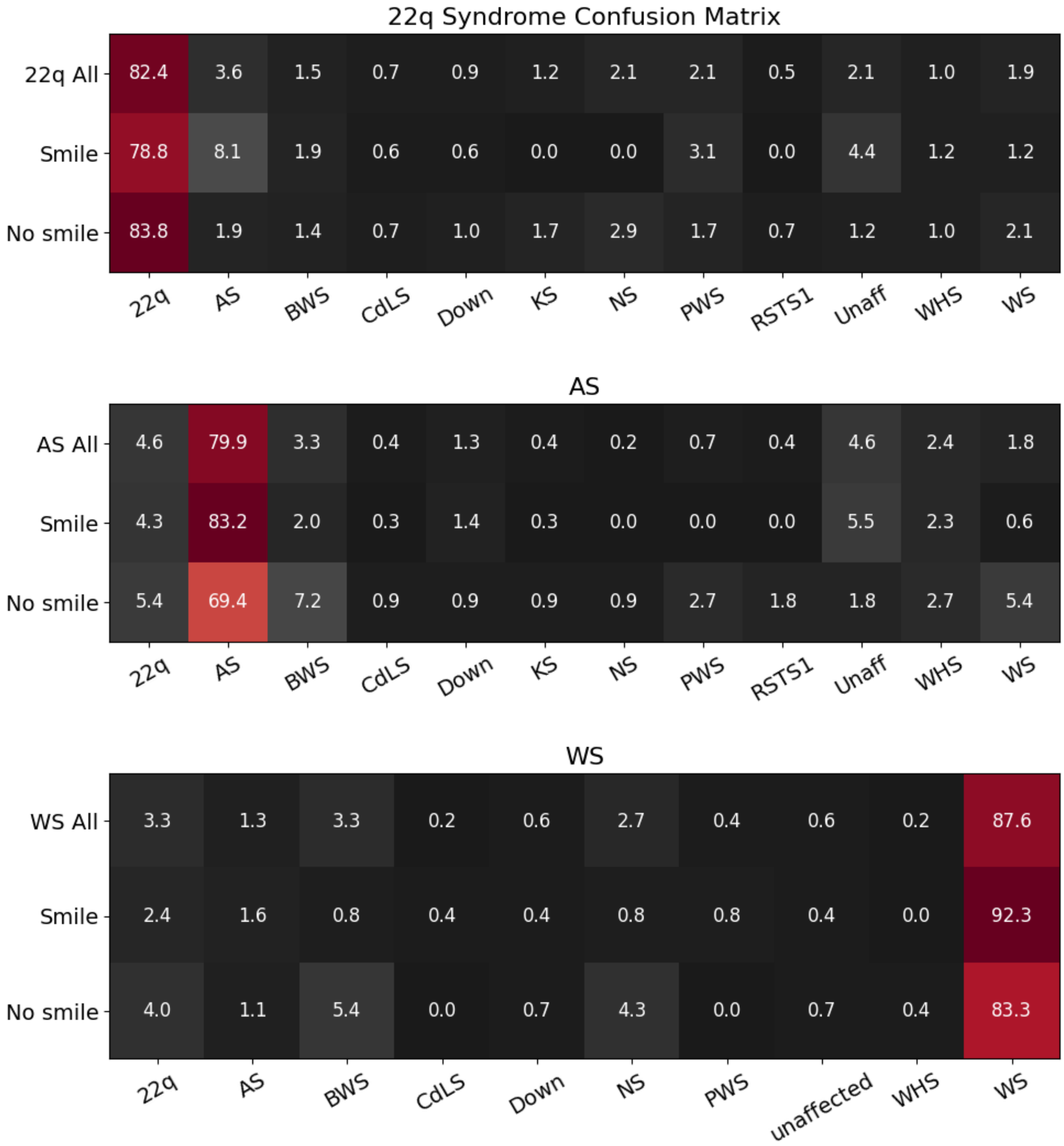

22q Syndrome Confusion Matrix
22q All
82.4 3.6 1.5 0.7 0.9 1.2 2.1 2.1 0.5 2.1 1.0 1.9
Smile
78.8 8.1 1.9 0.6 0.6 0.0 0.0 3.1 0.0 4.4 1.2 1.2
No smile
83.8 1.9 1.4 0.7 1.0 1.7 2.9 1.7 0.7 1.2 1.0 2.1
22q AS BWS CdLS Down KS NS PWS RSTS1 Unaff WHS WS
AS
AS All
4.6 79.9 3.3 0.4 1.3 0.4 0.2 0.7 0.4 4.6 2.4 1.8
Smile
4.3 83.2 2.0 0.3 1.4 0.3 0.0 0.0 0.0 5.5 2.3 0.6
No smile
5.4 69.4 7.2 0.9 0.9 0.9 0.9 2.7 1.8 1.8 2.7 5.4
22q AS BWS CdLS Down KS NS PWS RSTS1 Unaff WHS WS
WS
WS All
3.3 1.3 3.3 0.2 0.6 2.7 0.4 0.6 0.2 87.6
Smile
2.4 1.6 0.8 0.4 0.4 0.8 0.8 0.4 0.0 92.3
No smile
4.0 1.1 5.4 0.0 0.7 4.3 0.0 0.7 0.4 83.3
22q AS BWS CdLS Down NS PWS unaffected WHS WS

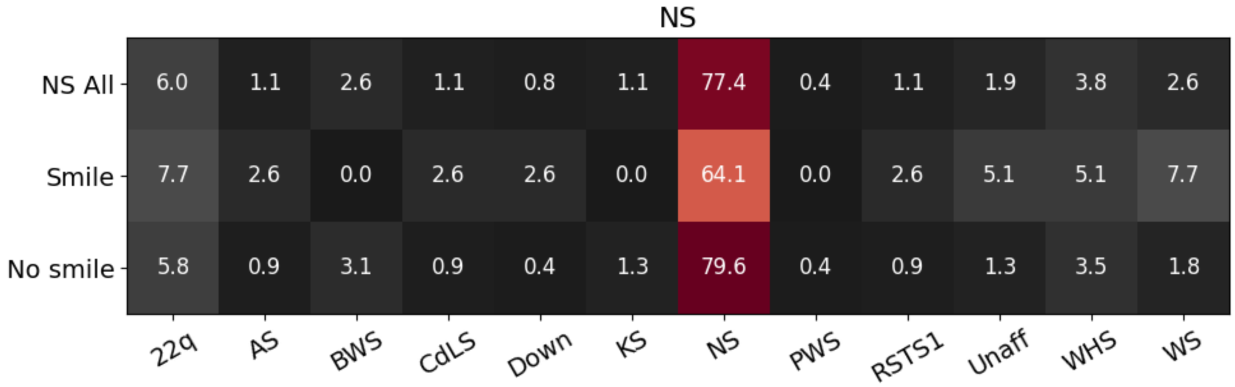

Supplementary Figure 2. Confusion matrices depicting prediction probabilities obtained through a five-fold cross-validation method using real images manually annotated as either "smile" or "no smile" in syndromic facial images. Within the matrices, columns portray the classifier's predicted labels, allowing for a comparison of accuracy between images with different facial expressions (smile or no smile).

		WS	Other Conditions for WS survey
**1**	**Overall accuracy**	72.80%	82.27%
**2**	**Smile**	82.44%	80.30%
**3**	**No Smile**	58.33%	83.81%

Supplementary Table 1. Clinical geneticist accuracy in assessing smiling versus non-smiling images of people with WS, compared to other control syndromic images. This table was computed based on the survey data from our previous work [1].

**Years of practice**	**<1 year**	**1-5 years**	**5-10 years**	**>10 years**	**Residents/fellows**
**Number of respondents**	3	7	10	34	2
**Percentage**	5%	12%	18%	61%	4%

**Number (percent) of participants rating the importance of facial features in clinical diagnosis**		
**Minor**	**Intermediate**	**Major**
6	28	22
11%	50%	39%

Supplementary Table 2. Characteristics of clinician participants. Respondents were board-certified or board-eligible clinical geneticists. The upper section indicates experience level; the lower section indicates how participations rated the importance of facial features in clinical diagnosis.

	**Survey 1**	**Survey 2**	**P-value**
**Avg Accuracy**	53.30%	52.00%	0.57533
**Avg Accuracy for reconstructed**	58.90%	55.70%	0.34611
**Avg Accuracy for expression-manipulated**	47.80%	48.30%	0.89012

Supplementary Table 3. Performance of the clinician participants over all the diseases. Two versions of the survey were used, allowing for a reconstructed image to be shown to one group and the corresponding expression-manipulated image to be shown to the other group. Each survey included 32 total images, with an equal number of reconstructed and expression-manipulated images.

**Comparison**	**Correlation**
All images: classifier vs geneticist	0.206
22q reconstructed: classifier vs geneticist	0.653
22q expression-manipulated: classifier vs geneticist	-0.752

22q all images: classifier vs geneticist	-0.015
AS reconstructed: classifier vs geneticist	0.093
AS expression-manipulated: classifier vs geneticist	-0.476
AS all images: classifier vs geneticist	-0.240
NS reconstructed: classifier vs geneticist	0.362
NS expression-manipulated: classifier vs geneticist	0.140
NS all images: classifier vs geneticist	0.257
WS reconstructed: classifier vs geneticist	0.509
WS expression-manipulated: classifier vs geneticist	0.447
WS all images: classifier vs geneticist	0.358
All reconstructed: classifier vs geneticist	0.443
All expression-manipulated only: classifier vs geneticist	0.014
All expression-manipulated no smile to smile	0.024
All expression-manipulated smile to no smile	-0.039

Supplementary Table 4. Correlation in performance between the model and clinical geneticists. Conditioned on the genetic condition and image type (i.e., reconstructed or expression-manipulated), we computed the correlation between the ground-truth predicted probabilities of the classifier and the average accuracy of the clinical geneticists. Overall, for the reconstructed images, the model and geneticists have moderate correlation (r=0.443). However, such correlation does not exist for the expression-manipulated images (r=0.014). This suggests that when the facial expressions are altered, the model and geneticists do not respond similarly. The most difference is observed for expression-manipulated images in 22q and AS (r=-0.752 and -0.476, respectively); here, over these images, when the model predicts an image to have more syndromic features (i.e., higher predicted probability for the ground-truth), then human geneticists see the opposite (and vice-versa).

References

[1]. Duong, D., et al., *Neural networks for classification and image generation of aging in genetic syndromes.* Frontiers in Genetics, 2022. **13**: p. 864092.
